# Dysbiosis in inflammatory bowel diseases: egg, not chicken

**DOI:** 10.3389/fmed.2024.1395861

**Published:** 2024-05-23

**Authors:** Eduard F. Stange

**Affiliations:** Klinik für Innere Medizin I, Universitätsklinik Tübingen, Tübingen, Germany

**Keywords:** Crohn’s disease, ulcerative colitis, microbiome, dysbiosis, intestinal barrier

## Abstract

There is agreement that inflammatory bowel diseases are, both in terms of species composition and function, associated with an altered intestinal microbiome. This is usually described by the term “dysbiosis,” but this is a vague definition lacking quantitative precision. In this brief narrative review, the evidence concerning the primary or secondary role of this dysbiotic state is critically evaluated. Among others, the following facts argue against a primary etiological impact: 1) There is no specific dysbiotic microbiome in IBD, 2) the presence or absence of mucosal inflammation has a profound impact on the composition of the microbiome, 3) dysbiosis is not specific for IBD but linked to many unrelated diseases, 4) antibiotics, probiotics, and microbiome transfer have a very limited therapeutic effect, 5) the microbiome in concordant twins is similar to disease-discordant twins, and 6) the microbiome in relatives of IBD patients later developing IBD is altered, but these individuals already display subclinical inflammation.

## 1 Introduction

Dysbiosis is not only a prolific topic but also poorly defined; at least there is no quantitative definition. The term dysbiosis is generally described “as an alteration in the ecosystem associated to pathology” (1). Dysbiosis may manifest not only as reduced diversity but also as relative or absolute alterations in the microbial composition (increases or decreases), the proliferation of pathobionts, and shifts in the functional capacities of the microbiome. Since even one of these alterations may indicate “dysbiosis,” the concept implied by this term is quite diffuse. Efforts to define more quantitative dysbiotic enterotypes have yielded considerable overlap (2). There are many diverse pathologies associated with different forms of intestinal dysbiosis: diabetes, obesity, depression, multiple sclerosis, and cardiovascular disease, in addition to IBD (3).

The major human IBDs, Crohn’s disease and ulcerative colitis, are well-defined diseases exhibiting a chronic mucosal or transmural, sometimes even extraintestinal, inflammatory state related to the mere presence of the intestinal microbiome (4). The commensal microbes and pathobionts may adhere to and invade the mucosa, and, as a consequence, antimicrobial antibodies and T-cell reactivity against bacteria appear early in the disease course (5–7). Surgical diversion of the intestinal contents has long been known to alleviate distal inflammation, and a limited benefit of fecal microbiome transfer has also been shown, at least for ulcerative colitis. The evidence for Crohn’s disease is less convincing (8, 9). However, with a few exceptions, such as pouchitis, antibiotics generally are not effective in IBD (10).

Explaining this bacterial microinvasion and the consequent immune response, there is extensive evidence of a defective mucosal barrier in both diseases (11, 12). It is linked to both genetics (13) and the environment (14) and appears to trigger the inflammation. The standard suspicion of an “overshooting immune response” may simply reflect a perfectly adequate defense against this intrusion.

In IBD, this mutually aggressive host/microbe interaction dysbiosis has been suggested to play a *primary* role in leading to pathology (15, 16), answering the chicken and egg question in favor of dysbiosis acting as the chicken. The present narrative minireview, after critically screening the data, might convince the reader of the opposite, i.e., the altered microbiome may indeed be *secondary* (arguments listed in Table 1). The focus is on human IBD, less on experimental models that may or may not reflect these diseases. The relevant literature was screened using the keywords Crohn’s disease and ulcerative colitis in PubMed.

**Table 1:** Arguments against dysbiosis being a primary etiological factor.

The term “dysbiosis” is ill-defined and there is no specific dysbiotic microbiome in IBD Conspicuously, the major difference between IBD and controls is the presence of mucosa-adherent bacteria The presence or absence of mucosal inflammation has a profound impact on the composition of the microbiome Clinical remission is often associated with a “normalization” of the gut microbiome Pathobionts have been proven to be associated with IBD but are not specific to IBD Dysbiosis is not specific to IBD but is linked to many unrelated diseases, including obesity, irritable bowel syndrome, cardiac, and neurological diseases Antibiotics, probiotics, and microbiome transfer have been shown to affect disease activity, but only in very defined clinical situations The microbiome in concordant twins is similar to disease-discordant twins The microbiome in relatives of IBD patients later developing IBD is altered but these individuals already display subclinical inflammation Most of the evidence points to the primary role of a barrier defect affecting the composition of the microbiome In this interaction, a vicious cycle is possible

## 2 Mucosal microbes as a trigger for inflammation

In pioneering study, Swidsinski et al. (5) found high concentrations of bacteria in the intestinal mucosa of patients with IBD but not in controls. The concentrations of bacteria increased progressively with the severity of disease in the inflamed and non-inflamed colon, and some bacteria were also found intracellularly in the mucosa, i.e., had invaded. They concluded that this observation was not secondary to inflammation and that “the healthy mucosa is capable of holding back fecal bacteria” (5). This capacity was apparently defective in IBD. In later studies, they described a biofilm containing particularly bacteroides species, approximately 100-fold more than a similar biofilm in irritable bowel syndrome (IBS) (17). Such a biofilm in cases with IBS and IBD was later confirmed (18).

The relevance of the intestinal microbiome in the pathogenesis of IBD was introduced in a seminal study by Sartor et al. (4). They had originally based their argument for the key role of the microbiome on gnotobiotic experimental animals because most, if not all, animal models of IBD depended on the luminal microbiome for inducing chemically or genetically mediated mucosal inflammation. In a complementary development, it was shown that antibodies known to be associated with IBD, such as ANCA’s or anti-cBir, are not autoimmune in the strict sense but directed primarily against bacterial structures (19), although the full spectrum is much more complex (20). Actually, some truly autoimmune antibodies directed against human tissues may well be initially induced by bacterial antigens with cross-reactivity.

Accordingly, dendritic cells loaded with bacteria stimulate an IgA secretion that limits the penetration of bacteria into the mucosa in the normal state (21, 22). This antibody response is joined and supported by a T-cell activation (TH1/TH17), which is also triggered by and directed against bacterial epitopes (23, 24), mostly described as “exaggerated.” Alternatively, this “overactivation,” which obviously leads to a breakdown of mucosal tolerance to enteric bacteria, may well be secondary to an abnormally massive bacterial invasion due to a primary barrier defect.

## 3 Dysbiosis in IBD, with and without inflammation

In 2004, Ott et al. (25) described a reduction in diversity of the colonic mucosa-associated bacterial microflora in patients with active inflammatory bowel disease. This drop by 50% compared with controls was mostly due to a loss of normal anaerobic bacteria such as *Bacteroides, Eubacterium,* and *Lactobacillus* species. Since all patients exhibited active inflammation, there was no non-inflamed IBD control. Using a metagenomic approach, this reduced diversity was essentially confirmed in the fecal microbiota of Crohn’s disease patients with respect to firmicutes such as *Clostridium leptum* and the *Bacteroides fragilis* subgroup (26). However, using a quantitative rather than a relative approach, some differences between Crohn’s disease and controls were lost (2). Another interesting species in this context is *Akkermansia,* which appears to be protective despite its role in mucin degradation (27).

Since further studies were inconsistent, Gevers et al. (28) focused on the treatment-naïve microbiome in new-onset Crohn’s disease, and although the diversity tended to be lower in IBD, there was considerable overlap with controls. They found an axis defined by an increased abundance in bacteria which included *Enterobacteriaceae, Pasteurellaceae, Veillonellaceae*, and *Fusobacteriaceae*, and decreased abundance in *Erysipelotrichales, Bacteroidales,* and *Clostridiales*, correlating strongly with disease severity (28). They also noted that certain metabolic functions, including carbohydrate, energy, lipid, and amino acid metabolism, as well as glycan biosynthesis capacities, were diminished in the Crohn’s disease microbiome. This functional aspect was then extended in a study on the functional disturbances in relapsing refractory Crohn’s disease (29). The study clearly demonstrated that there were fundamental differences between patients with active vs. quiescent disease. This aspect had already been emphasized already in a study comparing inflamed and non-inflamed regions, which concluded that the overall dysbiosis observed in inflammatory bowel disease patients relative to non-IBD controls might to some extent be a result of the disturbed gut environment rather than the direct cause of disease (30). Furthermore, a recent study concluded that colonic microbiota is associated with inflammation in IBD, although a residual difference vs. controls remained even in its apparent absence (31).

It has become evident that there is a complex interplay between the gut microbiota and host genetics (32), nutrition (33), and even something as “banal” as stool consistency (34). While these details are beyond the scope of this discussion, it is interesting to note that IBD risk genes alter the microbiome, even in healthy individuals without IBD (32). Finally, upon achieving remission, for example, by anti-TNF treatment, the distance to the healthy centroid of the microbiome is minimized, whereas it remains abnormal in non-remitters (35). Similarly, in ulcerative colitis, the principal components of the microbiome normalize after achieving long-term, but not short-term remission (36). In a very recent study, it appeared that microbial normalization upon achieving remission was much more pronounced following anti-TNF treatment compared to vedolizumab and ustekinumab treatment (16). Some studies, including the latter, found an association between the pre-therapy microbiome and therapy response but a solid prediction using the bacterial composition data alone is still not available (16). When taken together, a large part of the alterations observed in the IBD microbiome appear to be reversible and secondary to inflammation. At any rate, the details of this multi-omics interaction in this ecosystem are still not fully understood (37).

## 4 Pathobionts in IBD

A microbiota of low diversity may favor the outgrowth of a “symbiont that is able to promote pathology only when specific genetic and environmental conditions are altered in the host.” This is the definition of a pathobiont (38), and the most common is *E. coli LF83,* which has adherent/invasive properties (39). It may indeed survive in macrophages and have strong proinflammatory effects. However, this pathobiont is not specific to Crohn’s disease: in ileal specimens, AIEC strains were found in 21.7% of CD chronic lesions vs. in 6.2% of controls. In neoterminal ileal specimens, AIEC strains were found in 36.4% of CD early lesions (*p* = 0.034 vs. controls) and 22.2% of the healthy mucosa of CD patients. In colonic specimens, AIEC strains were found in 3.7% of CD patients, 0% of UC patients, and 1.9% of controls (39).

A potential counterpart is *Faecalibacterium prausnitzii* (40), which has been shown to be an anti-inflammatory commensal diminished in Crohn’s disease and ulcerative colitis. The overrepresentation of AIECs and the low counts of *faecalibacterium* may contribute to the inflammatory state, but, lacking specificity, both are unlikely to be causal in the strict sense. With these two exceptions, it is also worth noting that there is a lot of incongruency in various studies in the field at the species level (1). Recently identified new pathobiont candidates comprise *Clostridium innocuum, Atopobium parvulum, Ruminococcus gnavus, Bacteroides vulgatus*, and some others, but their truly pathogenic role is unconfirmed (41).

## 5 Dysbiosis is not specific for IBD

Another aspect of questioning the role of dysbiosis in IBD is the simple fact that this microbial disturbance has been described in a multitude of unrelated diseases (3). A prominent instance is obesity, where the gut microbiome may alter the intestinal barrier, gut-associated lymphoid tissues, induce insulin resistance, and increase food intake through interference with gastrointestinal peptides related to satiety (42). Both major types of diabetes, type I as well as type II, are associated with dysbiosis (43), and even its microvascular complications appear to be associated with the intestinal microbiome (44). There are also data linking celiac disease and cardiovascular diseases to the intestinal microbiome (45). Another review lists necrotizing enterocolitis, colorectal cancer, and *C. difficile*-associated enterocolitis as potential consequences of dysbiosis (3). Even various diseases with neuropathology are characterized by a dysbiotic intestinal microbiome (46). It may be argued that all of these represent different types of dysbiosis associated with a “leaky gut,” but none is accompanied by the massive inflammation typical for IBD.

## 6 Antibiotics, probiotics, and microbiome transfer in IBD

Antibiotics have been described as “deep modulators of gut microbiota between good and evil” (47). This implies that these compounds may, depending on their specificities, be harmful but sometimes even beneficial to a healthy microbiome. Considering the negative role as a risk factor of early antibiotic administration for IBD, the evidence of epidemiology warns against their unlimited use (48). Actually, specific antibiotics may increase the risk of flares in IBD (49). Although some specific IBD situations may benefit from antibiotics, including the postoperative state following ileal resection or pouchitis, no antibiotic has unequivocally been demonstrated to treat standard IBD. Some probiotics, such as *E. coli* Nissle, have clinical effects (50), but only in specific situations, such as the maintenance of remission in ulcerative colitis. It may be argued, however, that no antibiotic and no probiotic have been demonstrated to reverse the dysbiosis.

Indeed, the somewhat “messy” alternative of microbiome transfer to enhance diversity in IBD may be an option. In accordance with this idea, the donor microbiota richness and the number of transferred phylotypes were associated with treatment success (51). Accordingly, multi-donor studies were superior to single-donor designs (8). As a limitation of this approach, in most randomized controlled trials, remission was observed in only approximately 30% of patients (but superior to controls) (52), and the benefit of fecal microbiome transfer in Crohn’s disease has not been consistently observed (9, 53). At any rate, this therapy has shown that in some patients, a change in the microbiome (often not permanent) may impact disease activity, but this is not proof of dysbiosis causing the disease.

## 7 Microbiome in twins and other relatives

Early studies in twins already suggested that there were subtle differences between discordant twins, for example, the healthy twins exhibited more Lachnospiraceae and Ruminococcaceae than twins who were both healthy (54). A more recent and more detailed study revealed that the gut microbiome composition of healthy cotwins from IBD-discordant twin pairs displayed IBD-like signatures both on a species and pathway level (55). No differences were detected in the gut microbiome composition (beta-diversity) between healthy cotwins and IBD-twins, but both gut microbiomes differed from healthy controls. Thus, healthy discordant IBD twins live permanently with an IBD-like microbiome and most of them will stay healthy.

Another interesting series of studies stems from a large Canadian cohort of 3,483 IBD-patient relatives, 73 of whom developed IBD during the course of these investigations (56). Using a machine learning approach, they developed a microbiome risk score, yielding a modest hazard ratio of 2.2. This score “predicted” the (later) development up to 5 years before Crohn’s disease onset. The five most important taxa contributing to the MRS included *Ruminococcus torques, Blautia, Colidextribacter*, an uncultured genus-level group from *Oscillospiraceae*, and *Roseburia*. They found fecal calprotectin levels to reduce the hazard ratio to 1.42 but it was still statistically significant (*p* = 0.041). Since they found evidence of a non-linear effect of fecal calprotectin on CD risk, it is conceded by the authors that a proportion of healthy FDRs may already have had subclinical gut inflammation at the time of recruitment. Indeed, in prior studies in this cohort with later IBD, an antimicrobial antibody response, increased intestinal permeability, proteomic markers of subclinical inflammation and an association of the microbiome changes with the gut barrier had been described (57–59). Thus, it seems possible that even years before disease onset, the local milieu was abnormal, causing microbiome changes and bacterial translocations that stimulated an antibody response. As the authors concluded, experimental studies will be needed to assess whether the associations presented in this study represent a cause or effect of CD pathogenesis. However, fecal microbiome transfer from IBD-patients to healthy controls is, of course, unethical and, when it happened once by accident with a donor later developing Crohn’s disease, there was no IBD induced in the recipients (60).

## 8 Barrier vs. dysbiosis in a vicious cycle?

As discussed above, there is considerable evidence that dysbiosis is a secondary “epiphenomenon” related to disease activity but not *causally* related to IBD etiology. Most likely, the primary defect in IBD governing both the potential of microbial intrusion and microbiome composition is mucosal antimicrobial peptide (AMP) secretion (61) and local mucus production (14). All gastrointestinal surfaces synthesize several AMPs, such as defensins, cathelicidin, Reg3γ, lysozyme, and many others. This occurs in specialized Paneth cells of the small intestine, absorptive epithelial cells of the colon, and metaplastic Paneth cells. Many commensals exhibit relative resistance, which may explain their survival in the gut lumen and outer mucus layer, but only a few bacteria survive in the inner mucus layer (62, 63). In the healthy situation the outer mucus layer hosts a distinct intestinal microbial niche, different and separate from the lumen (62, 64). As mentioned above, even the protective inner layer is highly contaminated in IBD (5) because the basic defect in these diseases is most likely a decreased secretion of these peptides in the ileum (12) or inadequate induction in the colon of Crohn’s disease (61). In ulcerative colitis, these AMPs are adequately induced, but the mucus layer is defective in retaining these peptides (14).

These peptides, among other factors, control not only the ability of microbes to invade but also the microbial composition in the gut lumen. This apparently applies to defensins such as HD5 (65), whereas others, such as Reg3g, rather suppress local invasion with little effect in the lumen (66). It is Reg3γ that promotes the spatial separation between the epithelium and the microbes. Paneth cell-derived lysozyme defines the composition of the mucolytic microbiota and the inflammatory tone of the intestine (67). Furthermore, in humans, intestinal antibacterial gene expression is linked to bacterial composition (68), and both mucosal antibacterial response profile and fecal microbiota composition are linked to the disease progression in ulcerative colitis (69).

Recently, it has become evident that many of these peptides are enzymatically modified (70) or proteolytically degraded into a multitude of peptide fragments, some of which maintain antibiotic activity (71). These peptide fragments display defined specificities against various species and contribute to overall defense. The secretion of multiple AMPs determines the local milieu for the luminal and mucosal microbiomes, as well as regulating the intestinal microbiome, allowing for mutually beneficial cohabitation under normal conditions. There is now ample evidence that this homeostasis is disturbed in IBD (11–14). It seems at least plausible that inflammation-associated microbiome changes provide feedback on mucosal protective factors while also negatively impacting microbial composition.

Microbiota, on the other hand, control the secretion of antibacterial peptides (72) and mucins (73). Mucus not only forms a semisolid layer physically inhibiting bacterial invasion but also, through electrophilic charge interaction, retains AMP’s to form a chemical defense line (14). Furthermore, the expression of defensins and mucins is coordinated (74). Therefore, the real scenario is a close interplay and also the interdependency of the host defense and the microbiome in the gut (14, 75).

This is most apparent if the impact of both genetics and diet on the microbiome is focused. This impact may be mediated by internal factors, including AMPs, which are regulated by both genetics, including bacterial regulators such as NOD2 and ATG16L1, an autophagy gene, and diet (12). Thus, a fat-rich “Western” diet affects both Paneth cell function and the microbiome (76), suggesting that a part of these diet effects is not simply mediated by the “nutrient.” The multiple other mediators regulating defensins, such as smoking, known to be deleterious in Crohn’s disease have been reviewed previously (12). The other major effectors directing the production of both defensins and mucins are microbe-derived short-chain fatty acids and propionate (14), again in concordance with the concept of a tight interplay between microbes and mucosal defense. However, this control of the microbiome in the intestinal contents is not restricted to these players of innate immunity but also others, including T-cells (77). These, however, are also responsible for mucosal damage in IBD.

## 9 Conclusion

In conclusion, “dysbiosis” may represent a response of the gut microbiome to primary (partly genetic) alterations in mucosal antibacterial and mucus defense, rather than initially triggering the disease. However, with regard to the data implying a role in modulating disease activity (including fecal transfer), it is highly possible that a vicious cycle between dysbiosis and barrier occurs during the inflammatory process: some selected species may directly degrade mucus (78). It seems plausible that the barrier is the chicken and dysbiosis the egg, but after hatching all chicken will interact. Accordingly, we remain skeptical that the direct microbial approach using certain species, probiotics, or fecal microbiome transplants will effectively and permanently treat, or even heal, these chronic debilitating diseases (79, 80). Finally, it should be emphasized that fungi (81) and intestinal virome (82) add further complexity to the field but remain to be investigated more extensively in the future.

## Author contributions

EFS: Conceptualization, Writing – original draft, Writing – review & editing.
